# Brain immune cell composition and functional outcome after cerebral ischemia: comparison of two mouse strains

**DOI:** 10.3389/fncel.2014.00365

**Published:** 2014-11-19

**Authors:** Hyun Ah Kim, Stephanie C. Whittle, Seyoung Lee, Hannah X. Chu, Shenpeng R. Zhang, Zihui Wei, Thiruma V. Arumugam, Anthony Vinh, Grant R. Drummond, Christopher G. Sobey

**Affiliations:** ^1^Department of Pharmacology, Monash UniversityClayton, VIC, Australia; ^2^Department of Physiology, Yong Loo Lin School of Medicine, National University of SingaporeSingapore, Singapore; ^3^School of Pharmacy, Sungkyunkwan UniversitySuwon, South Korea; ^4^School of Biomedical Sciences, The University of QueenslandSt Lucia, QLD, Australia; ^5^Department of Surgery, Monash Medical Centre, Southern Clinical School, Monash UniversityClayton, VIC, Australia

**Keywords:** cerebral ischemia-reperfusion, immune cell infiltration, inflammation, middle cerebral artery occlusion, stroke, Th1/Th2 balance

## Abstract

Inflammatory cells may contribute to secondary brain injury following cerebral ischemia. The C57Bl/6 mouse strain is known to exhibit a T helper 1-prone, pro-inflammatory type response to injury, whereas the FVB strain is relatively T helper 2-prone, or anti-inflammatory, in its immune response. We tested whether stroke outcome is more severe in C57Bl/6 than FVB mice. Male mice of each strain underwent sham surgery or 1 h occlusion of the middle cerebral artery followed by 23 h of reperfusion. Despite no difference in infarct size, C57Bl/6 mice displayed markedly greater functional deficits than FVB mice after stroke, as assessed by neurological scoring and hanging wire test. Total numbers of CD45^+^ leukocytes tended to be larger in the brains of C57Bl/6 than FVB mice after stroke, but there were marked differences in leukocyte composition between the two mouse strains. The inflammatory response in C57Bl/6 mice primarily involved T and B lymphocytes, whereas neutrophils, monocytes and macrophages were more prominent in FVB mice. Our data are consistent with the concept that functional outcome after stroke is dependent on the immune cell composition which develops following ischemic brain injury.

## Introduction

Stroke is the 4th leading cause of death after heart disease, cancer and chronic lower respiratory disease, and over a third of survivors are left with major neurological injury (Go et al., 2013). Approximately 85% of stroke cases are of the ischemic type (Go et al., 2013), in which an embolus or local thrombus causes occlusion of a major cerebral artery and results in disruption of brain blood flow. Whilst thrombolysis by intravenous recombinant tissue plasminogen activator (rt-PA) may be effective in improving outcome by promoting reperfusion, it has a number of limitations, including a short therapeutic window of 3–4.5 h (<10% of stroke patients receive rt-PA) (Gravanis and Tsirka, 2008). For further advances in the clinical treatment of ischemic stroke, the complex mechanisms of cellular injury following cerebral ischemia must be elucidated to provide novel targets for future therapies.

It is now established that the initial insult in ischemic stroke is followed by induction of cytokines and chemokines, which attract numerous inflammatory cell types to the damaged brain region, which ultimately contribute to secondary brain injury (Gelderblom et al., 2009; Chu et al., 2014). Growing evidence indicates the importance of T lymphocytes in cerebral ischemic damage, whereby they become activated and infiltrate the brain within 24 h (Yilmaz et al., 2006; Hurn et al., 2007; Urra et al., 2009a), although the mechanism(s) underlying their actions are not fully clear. Recombination activating gene 1-deficient mice, which lack T and B lymphocytes, have less severe brain injury following cerebral ischemia, and this protection is lost upon reconstitution with T but not B lymphocytes (Yilmaz et al., 2006; Kleinschnitz et al., 2010).

T lymphocytes (CD3^+^ cells) are mostly comprised of CD4^+^ T helper (Th) and CD8^+^ cytotoxic T (Tc) cell subpopulations, both of which are thought to play detrimental roles in ischemic stroke (Yilmaz et al., 2006). There is also evidence that regulatory T lymphocytes (Tregs; CD4^+^CD25^+^FoxP3^+^) may modulate the severity of stroke outcome (Liesz et al., 2009), despite exerting acutely detrimental effects by promoting intravascular coagulation during reperfusion (Kleinschnitz et al., 2013). Major distinct Th cell types include Th1 and Th2, and are defined according to the cytokines they release (Abbas et al., 1996). In general terms, Th1 cells promote an inflammatory response through secretion of pro-inflammatory cytokines [e.g., interleukin(IL)-2, IL-12, interferon(IFN)-γ, and tumor necrosis factor(TNF)-α], whereas Th2 cells promote a humoral or allergic response by secretion of anti-inflammatory cytokines (e.g., IL-4, IL-10, and IL-13) (Arumugam et al., 2005; Jin et al., 2010).

Clarification of the importance of Th1 and Th2 immunity in acute stroke is needed to define the complex evolution of cerebral ischemic injury and potentially identify therapeutic strategies to limit stroke injury. Here, we have studied representative mouse strains commonly accepted as Th1-dominant (C57Bl/6) and Th2-dominant (FVB) (Whitehead et al., 2003) to investigate stroke outcome in a prototypical Th1- or Th2-prone immune environment, respectively.

## Materials and methods

### Animals

This study fully adheres to the Animal Research: Reporting *In Vivo* Experiments (ARRIVE) guidelines (Kilkenny et al., 2010). All animal experiments were conducted in accordance with National Health and Medical Research Council of Australia guidelines for the care and use of animals in research and approved by the Monash University Animal Ethics Committee (Projects SOBSB/2010/10 and SOBSB/2011/112). A total of 201 male mice (C57Bl/6: *n* = 89, 19–33 g; FVB: *n* = 112, 25–39 g) aged 8–15 weeks were studied. The mice had free access to water and food pellets before and after surgery. Thirty-seven mice were excluded from the study because they: (1) died during surgical procedure (C57Bl/6: *n* = 12; FVB: *n* = 24) or (2) were euthanized prior to 24 h as per institutional ethics requirements due to severe functional impairment (C57Bl/6: *n* = 1).

### Transient focal cerebral ischemia

Focal cerebral ischemia was induced by transient intraluminal filament occlusion of the right middle cerebral artery (MCA) as described previously (Jackman et al., 2009; Brait et al., 2010). Mice were anesthetized with ketamine-xylazine (80 and 10 mg/kg, respectively; intraperitoneal). Rectal temperature was monitored and maintained at 37.5 ± 0.5°C throughout the procedure and until animals regained consciousness using an electronic temperature controller (Testronics, Kinglake, Victoria, Australia) linked to a heat lamp. The right proximal common carotid artery was clamped, and a 6–0 nylon monofilament with silicone-coated tip (Doccol Co., Redlands, CA, USA) was inserted and gently advanced into the distal internal carotid artery, 11–12 mm distal to the carotid bifurcation, occluding the MCA at the junction of the Circle of Willis. Severe (~80%) reduction in regional cerebral blood flow (rCBF) was confirmed using transcranial laser-Doppler flowmetry (Perimed, Järfälla, Sweden) in the area of cerebral cortex supplied by the MCA. The filament was then tied in place and the clamp was removed. After 1 h of cerebral ischemia, the monofilament was retracted to allow reperfusion for 23 h. Reperfusion was confirmed by an immediate increase in rCBF, which reached the pre-ischemic level within 5 min. The wound was then closed and the animal was allowed to recover. Regional CBF was recorded for 30 min after the induction of reperfusion. Sham-operated mice were anesthetized and the right carotid bifurcation was exposed, dissected free from surrounding connective tissue but no filament was inserted. All animals were administered 1 mL of sterile saline via a subcutaneous injection for rehydration after surgery.

### Neurological assessment

At the end of the experiment (24 h after induction of stroke/sham surgery), neurological assessment was performed using a modified six-point scoring system (Jackman et al., 2009; Brait et al., 2010): 0, normal motor function; 1, flexion of torso and contralateral forelimb when mouse is lifted by the tail; 2, circling when mouse held by the tail on a flat surface; 3, leaning to the one side at rest; 4, no spontaneous motor activity; 5, death within 24 h. A hanging wire test was also performed in which mice were suspended from a wire 30 cm high for up to 180 s, and the average time of 3 trials with 5-min rest periods in between was recorded. Neurological assessment was evaluated by an observer blinded to experimental groups.

### Cerebral infarct and edema volumes

Mice were killed at 24 h by inhalation of isoflurane, followed by decapitation. The brains were immediately removed and snap frozen with liquid nitrogen. Coronal sections (30 μm) separated by ~420 μm were obtained and stained with thionin (0.1%) to delineate the infarct. Images of the sections were captured with a CCD camera mounted above a light box. Infarct volume was quantified as described previously (Jackman et al., 2009; Kim et al., 2012) using image analysis software (ImageJ, NIH, Bethesda, MD, USA), and corrected for brain edema, estimated using the following formula: corrected infarct volume = [left hemisphere area − (right hemisphere area − right hemisphere infarct area) × (thickness of section + distance between sections)] (Tsuchiya et al., 2003; Xia et al., 2006). Edema-corrected infarct volumes of individual brain sections were then added giving a three-dimensional approximation of the total infarct volume. Total, cortical and subcortical infarct volumes were quantified individually.

### Gross cerebrovascular anatomy

For gross comparison of cerebrovascular anatomy, some naïve animals (*n* = 3 of each strain, without any surgical procedures) were deeply anesthetized by inhalation of isoflurane, the thorax was opened and intracardial perfusion was performed with PBS, followed by 4% paraformaldehyde and finally 4% Evans blue solution in 20% gelatin.

### Flow cytometry

On each occasion when flow cytometry was utilized, we studied at least one post-stroke mouse together with a time-matched sham-operated control mouse of each strain. Animals were euthanized at 24 h by inhalation of isoflurane, followed by blood removal by cardiac puncture and the whole mouse was then intracardially perfused with phosphate-buffered saline (PBS) and brain, blood, and spleen were collected. Leukocytes were purified from blood using red blood cell lysis buffer (155 mmol/L NH_4_Cl, 10 mmol/L KHCO_3_, and 3 mmol/L EDTA). Spleens were mechanically dissociated and passed through 70 μm nylon cell strainers (BD Falcon, Bedford, MA, USA) to obtain a single-cell suspension. Cells were then lysed with red blood cell lysis buffer and washed with PBS containing 1% bovine serum albumin. The brain was removed from the skull and after removing the cerebellum and olfactory bulb, was separated into left (contralateral) and right (ischemic) hemispheres. Each hemisphere was dissociated mechanically in digestion buffer containing collagenase type XI (125 U/mL), hyaluronidase (60 U/mL), and collagenase type I-S (450 U/mL) in Ca2^+^/Mg2^+^-supplemented PBS (Sigma, St Louis, MO, USA), and incubated at 37°C for 30 min with gentle agitation. The mixture was then passed through 70 μm nylon cell strainers to obtain a single-cell suspension. After washing with PBS (1200 rpm, 10 min), the cell pellet was resuspended in 3 mL 30% percoll (GE Healthcare, Uppsala, Sweden), underlaid with 70% percoll, and centrifuged for 20 min at 2400 rpm at room temperature without the use of a brake. The cells at the interphase of two density gradients were collected and washed with PBS containing 1% bovine serum albumin (1200 rpm, 10 min) for staining. All cells were incubated with appropriate antibodies listed in Table 1 at 4°C in darkness for 20 min. After staining, cells were analyzed by LSRII flow cytometer (BD Biosciences, Franklin Lakes, NJ, USA) and FlowJo software (Tree Star Inc., Ashland, OR, USA). Countbright counting beads (Invitrogen, Carlsbad, CA, USA) were included to define the absolute number of cells in the samples.

**Table 1:** **Summary of antibodies used for flow cytometry**.

**Antigen**	**Host/Isotype**	**Supplier**
CD8a-APC	Rat IgG2a, kappa	BD Phamingen
CD19-PE	Rat IgG2a, kappa	BioLegend
CD11c-Brilliant Violet 570	ArHam IgG	BioLegend
CD25-PE-Cy7	Rat IgG1, lambda	BioLegend
CD4-FITC	Rat IgG2b, kappa	BioLegend
CD45-APC-Cy7	Rat IgG2b, kappa	BioLegend
CD49b-PE	ArHam IgG	BioLegend
CD90.2-PE	Rat IgG2b, kappa	BioLegend
Ly6C-FITC	Rat IgG2c, kappa	BioLegend
Ly6G-PE-Cy7	Rat IgG2a, kappa	BioLegend
NK1.1-PE	Mouse IgG2a, kappa	BioLegend
CD11b-eFluro450	Rat IgG2b, kappa	eBioscience
CD3-eFluro450	Rat IgG2b, kappa	eBioscience
F4/80-APC	Rat IgG2a, kappa	eBioscience
7-Amino-actinomycin D (7AAD)		Invitrogen

### Gating strategy

Single cells were identified by forward scatter, and dead cells (7-amino actinomycin D^+^) were excluded. Cells were gated for CD45^+high^ and CD45^+med^ populations as described previously (Chu et al., 2014). CD45^+high^ populations were then divided into lymphoid cells, which include: B cells (B220^+^), T cells (CD49b^+^CD90^−^NK1.1^−^), thymocytes (CD49b^−^CD90^+^NK1.1^−^), NK cells (CD49b^+^CD90^+^NK1.1^+^), and NKT cells (CD49b^+^CD90^−^NK1.1^+^); and myeloid cells (CD11b^+^). CD45^+med^CD11b^+^F4/80^+^ cells were considered microglia. Two panels of antibodies were used, one of which was employed with each animal. Panel 1 enabled the counting of microglia and myeloid-derived leukocytes (i.e., CD11b^+^ cells), which include: neutrophils (Ly6G^+^), monocytes (Ly6C^+^), macrophages (F4/80^+^), and dendritic cells (CD11c^+^); whereas Panel 2 divided lymphocytes into: B cells (CD19^+^) and T cells (CD3^+^). T cells were then further subdivided into CD4^+^ T cells, CD8^+^ T cells, CD4^−^CD8^−^ T cells, and CD4^+^CD25^+^ T cells. Fluorescence-minus-one were included as negative controls to define positive populations for F4/80, CD11c, Ly6C, CD19, CD3, and CD25.

### Cytokine measurement

Single-cell suspensions of brain, blood and spleen were obtained as described for flow cytometry. All cells were resuspended in complete RPMI 1640 media supplemented with heat inactivated fetal bovine serum (10% w/v), streptomycin and penicillin (100 U/mL), L-glutamine (1%) and 2-mercaptoethanol (50 mM). Blood and spleen cells were seeded at 200,000 cells/well in a 96-well plate coated with anti-CD3. All cells in the brain hemisphere were seeded. Blood, spleen, and contralateral brain hemisphere of sham-operated mice was seeded as unstimulated controls. Recombinant mouse IL-2 (20 ng/mL) was added and cells were incubated for 48 h at 37°C in a humidified atmosphere of 5% CO_2_ in air. After stimulation, cells were spun down (15,000 rpm, 5 min) and supernatant was collected. Samples were analyzed for 7 key inflammatory cytokines (IL-4, IL-6, IL-10, IL-17A, IFN-γ, and TNF-α) using Cytometric Bead Array Mouse Th1/Th2/Th17 Cytokine kit (BD Biosciences, Franklin Lakes, NJ, USA). Samples and standards were prepared according to manufacturer's protocol. After adding capture beads, cells were analyzed by LSRII flow cytometer (BD Biosciences, Franklin Lakes, NJ, USA) and FCAP Array software (BD Biosciences, Franklin Lakes, NJ, USA).

### Statistical analysis

Values are presented as mean ± standard error. Results of the hanging wire test, infarct volume and flow cytometry, comparing C57Bl/6 and FVB sham- or stroke-operated mice, were analyzed using one-way analysis of variance with Bonferroni *post-hoc* test with selected multiple comparisons or a Student's unpaired *t*-test, as appropriate. The neurological deficit score was expressed as the median result per group and was analyzed using a Kruskal-Wallis test with Dunn's *post-hoc* test. A *P* value <0.05 was considered statistically significant. Statistical analyses were carried out using GraphPad Prism (GraphPad Software Inc, La Jolla, CA, USA).

## Results

### Cerebral blood flow profile and mortality

**Figure 1:**
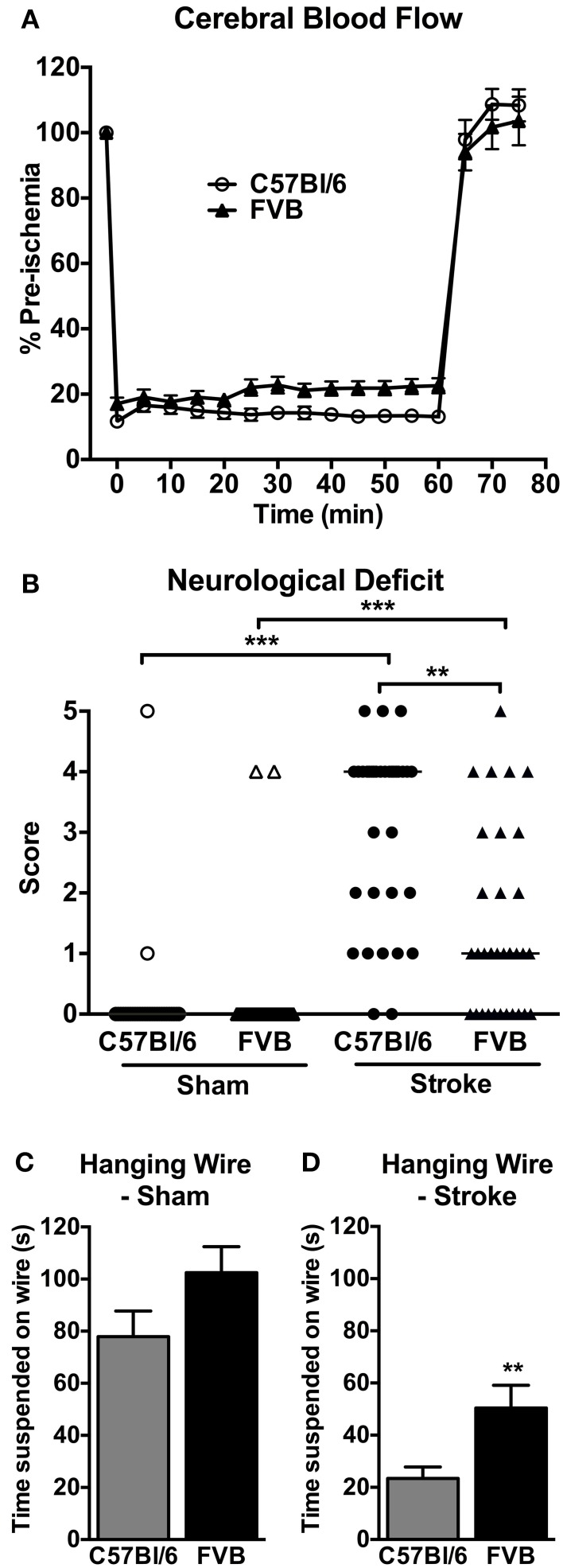
**(A)** Regional cerebral blood flow during 1 h middle cerebral artery occlusion (*n* = 30–31); (B) neurological deficit (*n* = 29–34; ^**^P < 0.01, ^***^P < 0.001; Kruskal-Wallis with Dunn's post-tests) and (C,D) hanging wire performance (*n* = 28–33; ^**^P < 0.01; Student's unpaired *t*-test) at 24 h after cerebral ischemia. There was less functional impairment in FVB mice compared to C57Bl/6 mice. Data are mean ± s.e.m. in **(A,C,D)**, and are median scores in **(B)**.

CBF was similarly reduced by ~80% in both C57Bl/6 and FVB mice following insertion of the monofilament (Figure 1A). No significant differences in CBF profiles were observed between C57Bl/6 and FVB mice. Mortality rates at 24 h after cerebral ischemia were 8.8% (3/34) and 2.9% (1/34) in C57Bl/6 and FVB mice, respectively (Figure 1B). Mice of both strains had no significant differences in the cerebrovascular anatomy; in particular, the posterior communicating arteries were present in both strains (Supplementary Figure 1).

### Neurological function

Mice of both strains typically had no neurological deficit (score of 0) at 24 h after sham surgery (Figure 1B). Following cerebral ischemia, both C57Bl/6 and FVB mice had significant neurological deficit compared to sham-operated mice of the same strain, although C57Bl/6 mice had greater deficit than FVB mice. Similarly, in the hanging wire test sham-operated C57Bl/6 and FVB mice achieved comparable hanging times (Figure 1C), whereas FVB mice achieved ~2-fold longer hanging times than C57Bl/6 mice at 24 h following cerebral ischemia (Figure 1D).

### Infarct and edema volumes

**Figure 2:**
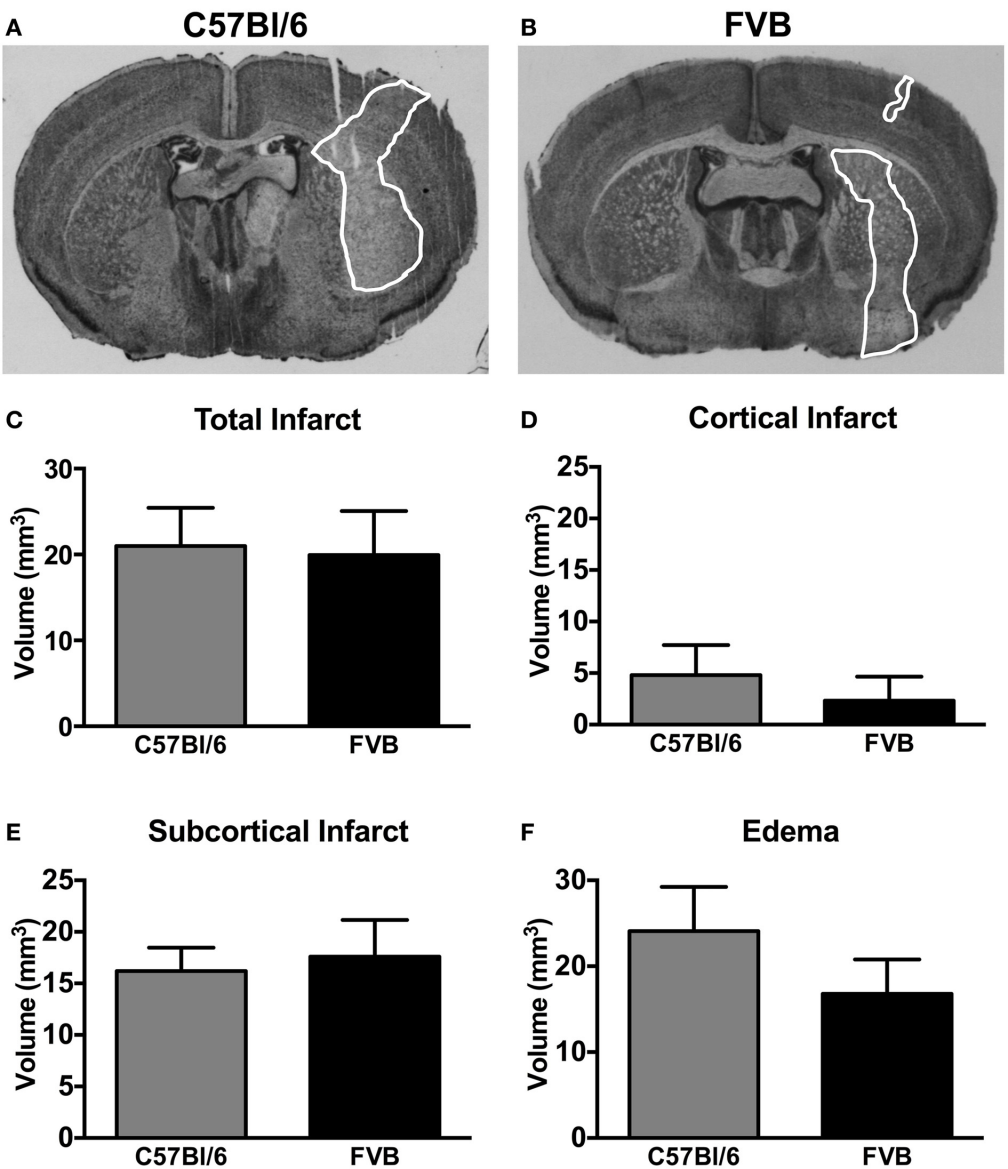
**Representative coronal brain sections from a (A) C57Bl/6 mouse and a (B) FVB mouse 24 h after 1 h middle cerebral artery occlusion (infarct areas are outlined in white)**. Infarct [**(C)**, total; **(D)**, cortical; **(E)**, subcortical] and **(F)** edema volumes are also shown (*n* = 11 per group; Student's unpaired *t*-test). There was no difference in the infarct volume between two strains of mice. Data are mean ± s.e.m.

Representative coronal sections of C57Bl/6 and FVB brains at 24 h after MCA occlusion are shown in Figures 2A,B, respectively. C57Bl/6 and FVB mice had similar total infarct (Figure 2C), cortical infarct (Figure 2E), subcortical infarct (Figure 2F) and edema (Figure 2D) volumes.

### Leukocyte infiltration in the brain

**Figure 3:**
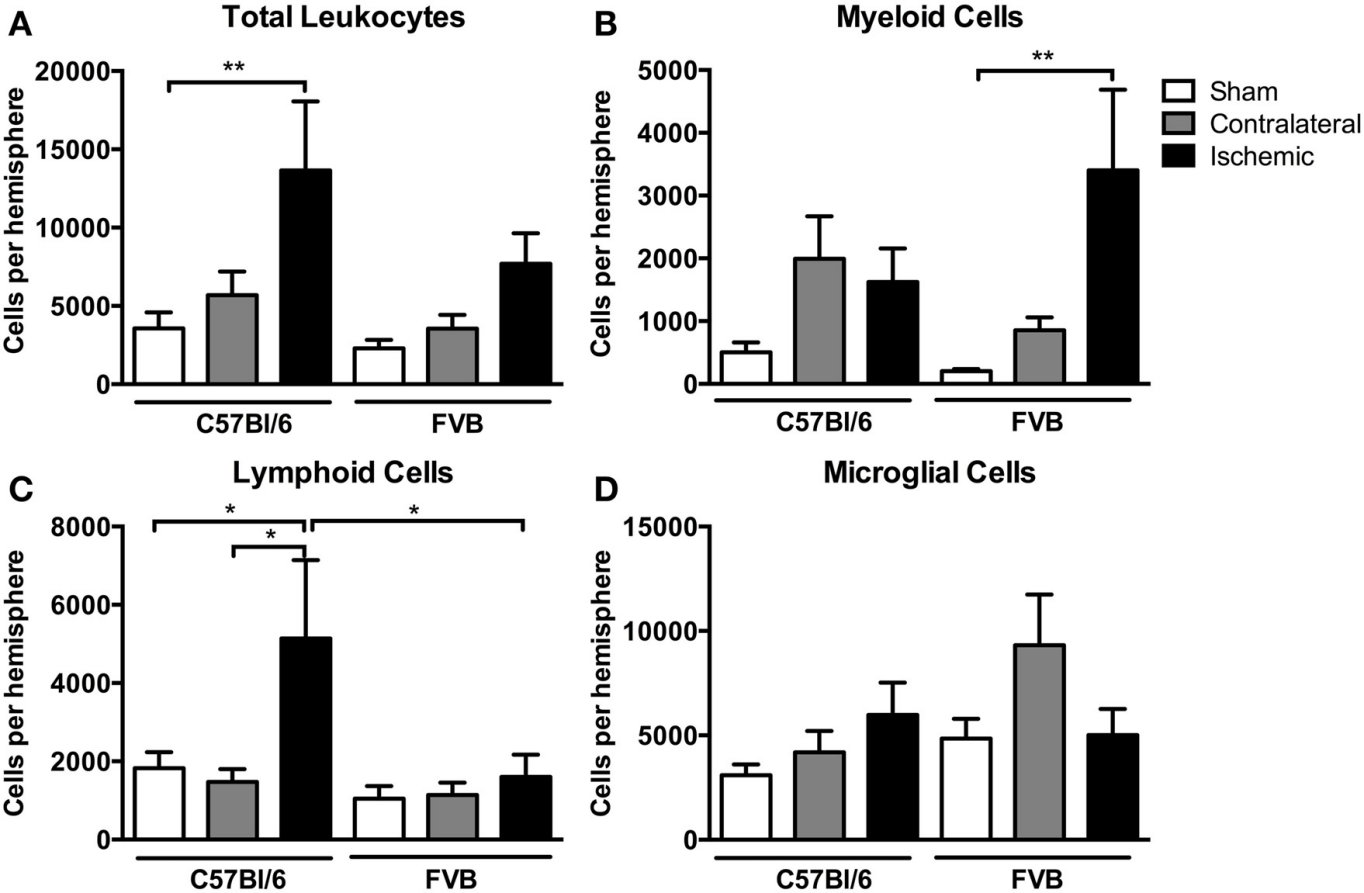
**Flow cytometric quantification of (A) total leukocytes and (B–D) leukocyte subsets in the brain at 24 h after 1 h middle cerebral artery occlusion**. Data are shown for the contralateral and ischemic hemispheres, and compared with sham control mice of the same strain (*n* values: total leukocytes *n* = 13–16, subsets *n* = 6–9; ^*^P < 0.05, ^**^P < 0.01; One-Way ANOVA with Bonferroni post-tests). There was a greater number of leukocytes, predominantly lymphoid cells, infiltrating the brain of C57Bl/6 mice compared to FVB mice. Data are mean ± s.e.m.

There was a ~4-fold increase in the total number of leukocytes in the ischemic hemisphere of C57Bl/6 mice compared to sham-operated mice (*P* < 0.01, Figure 3A). There also tended to be an increase in total leukocytes following ischemia in FVB mice, but there were ~40% fewer leukocytes than in C57Bl/6 mice (Figure 3A). Myeloid cells (CD11b^+^), comprising neutrophils (Ly6G^+^), dendritic cells (CD11b^+^CD11c^+^), macrophages (F4/80^+^) and monocytes (Ly6C^+^), were increased by a ~15-fold in the ischemic hemisphere of FVB mice to levels that were twice those in C57Bl/6 mice (*P* < 0.01, Figure 3B). By contrast, lymphoid cells were increased by 2–3-fold following ischemia in C57Bl/6 mice, whereas there was no change in lymphoid cell numbers in the brains of FVB mice following stroke (Figure 3C). No significant effect of stroke was observed in the number of microglia (CD45^+med^CD11b^+^F4/80^+^) in either strain (Figure 3D).

**Figure 4:**
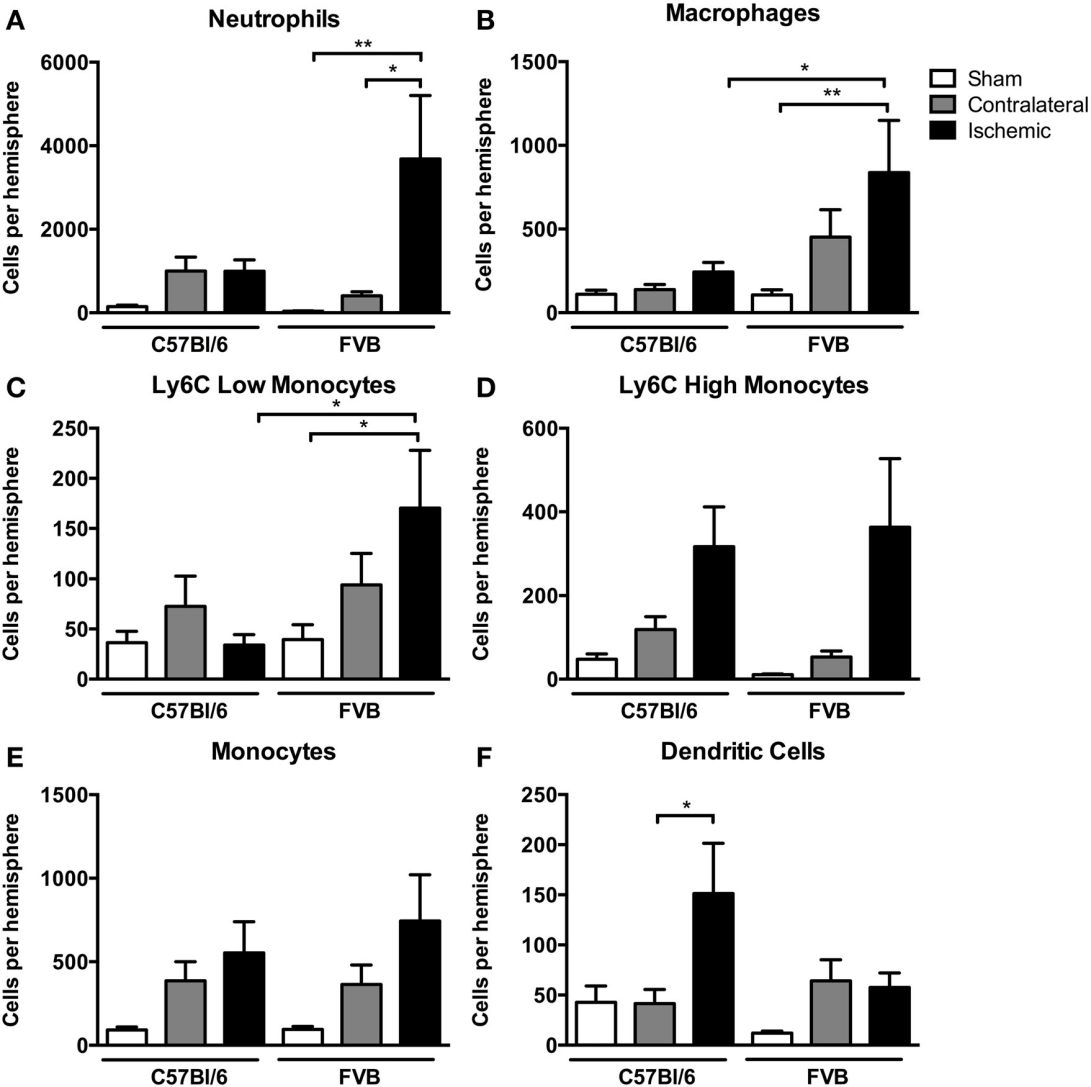
**Quantification of myeloid cell subpopulations [**(A)**, neutrophils; **(B)**, macrophages; **(C)**, Ly6C low monocytes; **(D)**, Ly6C high monocytes; **(E)**, monocytes; **(F)**, dendritic cells] in the brain 24 h after 1 h middle cerebral artery occlusion**. Data are shown for the contralateral and ischemic hemispheres, and compared with sham control animals of the same strain (*n* = 6–9; ^*^P < 0.05, ^**^P < 0.01; One-Way ANOVA with Bonferroni post-tests). Data are mean ± s.e.m.

Among myeloid cells, neutrophils were most prevalent, with these cells being ~4-fold more numerous in ischemic hemispheres of FVB than in C57Bl6 mice (Figure 4A). There was a similar profile of both macrophage and Ly6C^low^ monocyte numbers in the ischemic brain following stroke, with also ~4-fold more of these cells in FVB than C57Bl/6 mice (Figures 4B,C). Ly6C^high^ monocytes and total monocytes were present in similar numbers in the ischemic brains of the two strains (Figures 4D,E). In contrast, the number of dendritic cells in the ischemic hemisphere was ~3-fold higher in C57Bl/6 than FVB mice (Figure 4F).

**Figure 5:**
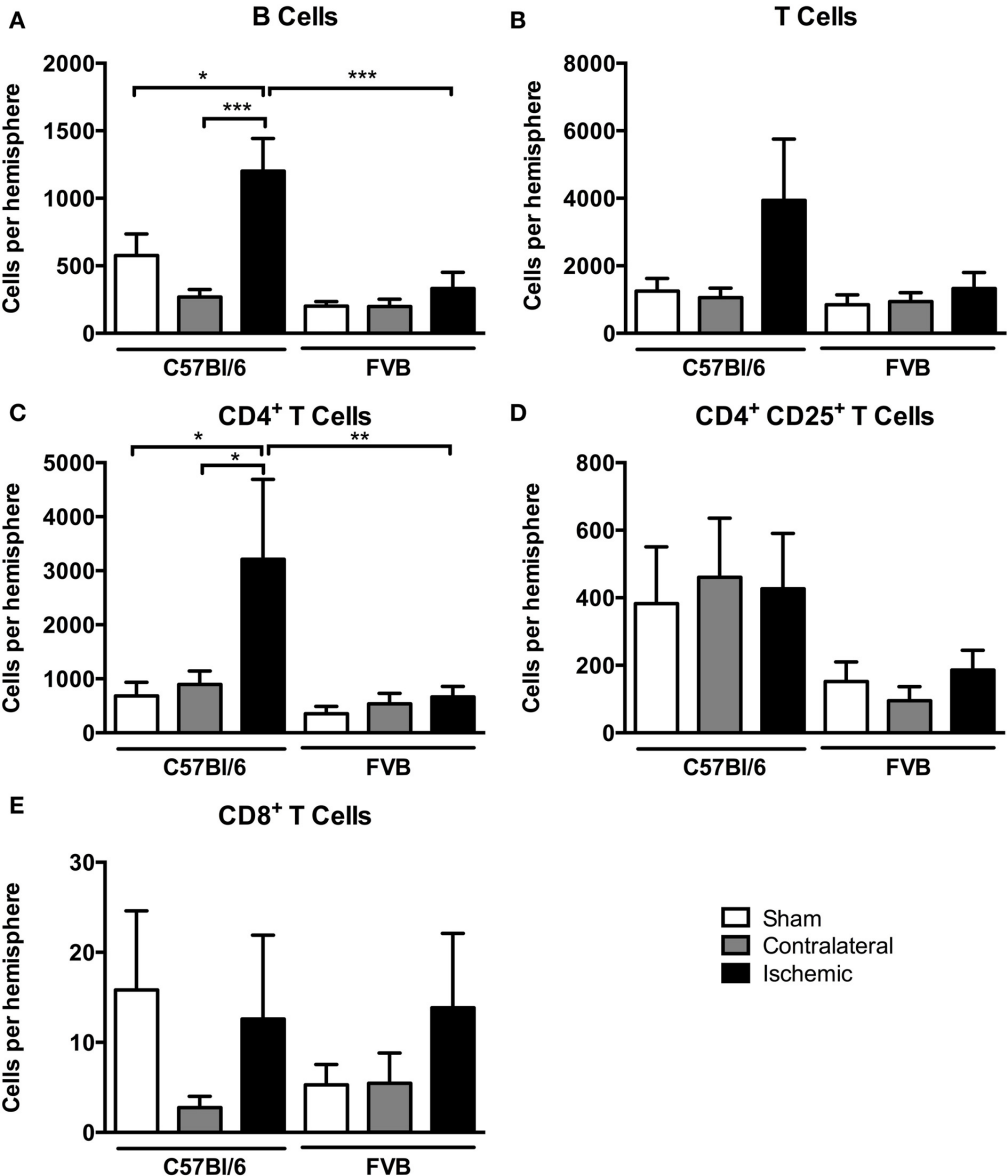
**Quantification of lymphocyte subpopulations [**(A)**, B cells; **(B)**, T cells; **(C)**, CD4^+^ T cells; **(D)**, CD4^+^ CD25^+^ T cells; **(E)**, CD8^+^ T cells] in the brain 24 h after 1 h middle cerebral artery occlusion**. Data are shown for the contralateral and ischemic hemispheres, and compared with sham control animals of the same strain (*n* = 4–8; ^*^P < 0.05, ^**^P < 0.01, ^***^P < 0.001; One-Way ANOVA with Bonferroni post-tests). Data are mean ± s.e.m.

Among lymphoid cells, there were marked increases in numbers of both B cells and T cells in the post-ischemic brain of C57Bl/6 mice but not FVB mice (Figures 5A,B). Further analysis of T cell subpopulations indicated that the increase in C57Bl/6 mice was mostly due to infiltration of CD4^+^CD25^−^ (“T helper”) cells and not to CD8^+^ (“cytotoxic”) nor CD4^+^CD25^+^ T cells, which includes Tregs (Figures 5C–E).

**Figure 6:**
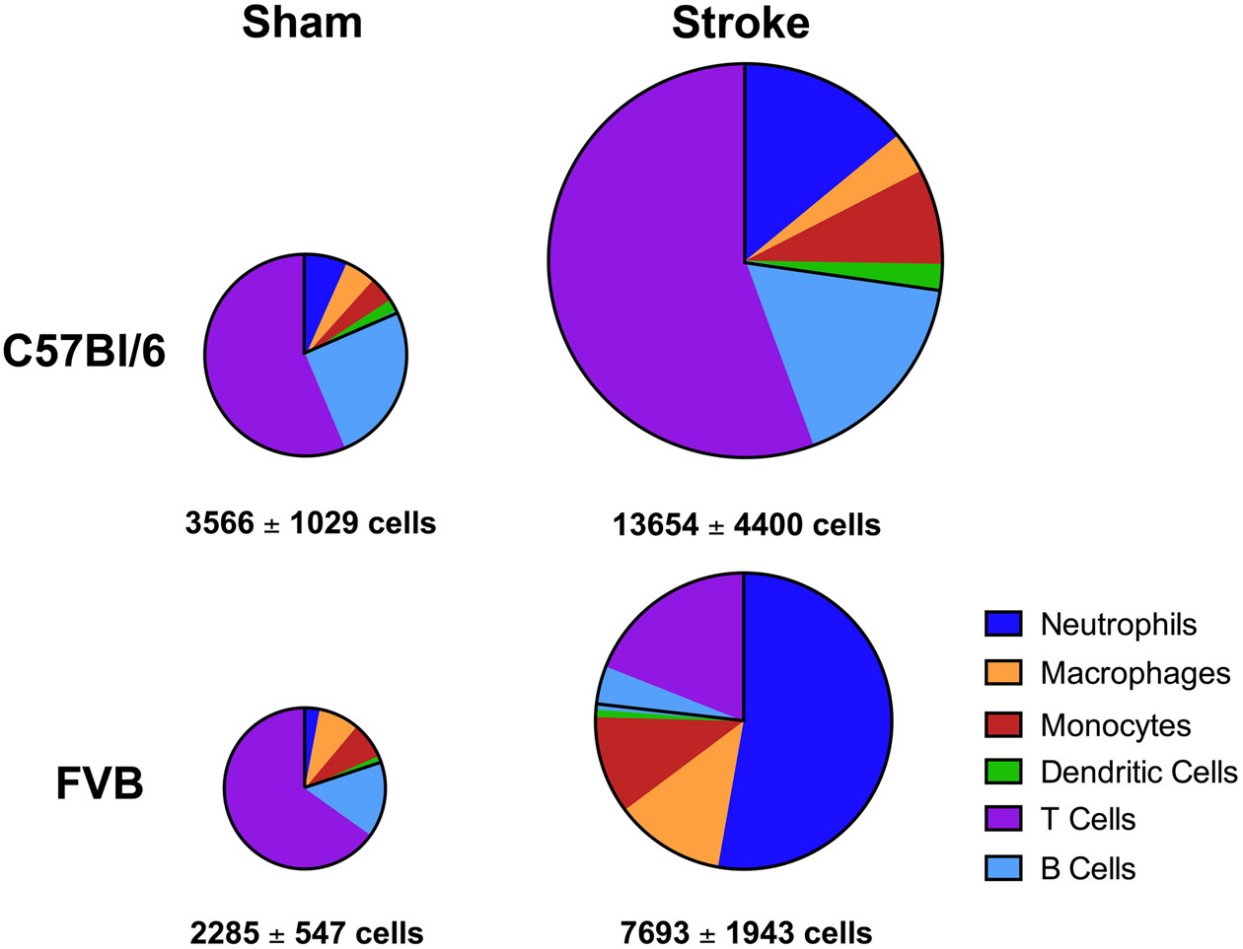
**Pie charts summarizing number and composition of immune cells in ischemic hemispheres of C57Bl/6 and FVB mice at 24 h after 1 h middle cerebral artery occlusion**. The area of each pie is proportional to the number of leukocytes per hemisphere (also shown as mean ± s.e.m.; total leukocytes *n* = 13–16, myeloid cells *n* = 6–9, lymphocytes *n* = 4–8). There was a greater number of leukocytes, predominantly lymphoid cells, infiltrating the brain of C57Bl/6 mice compared to FVB mice.

**Figure 7:**
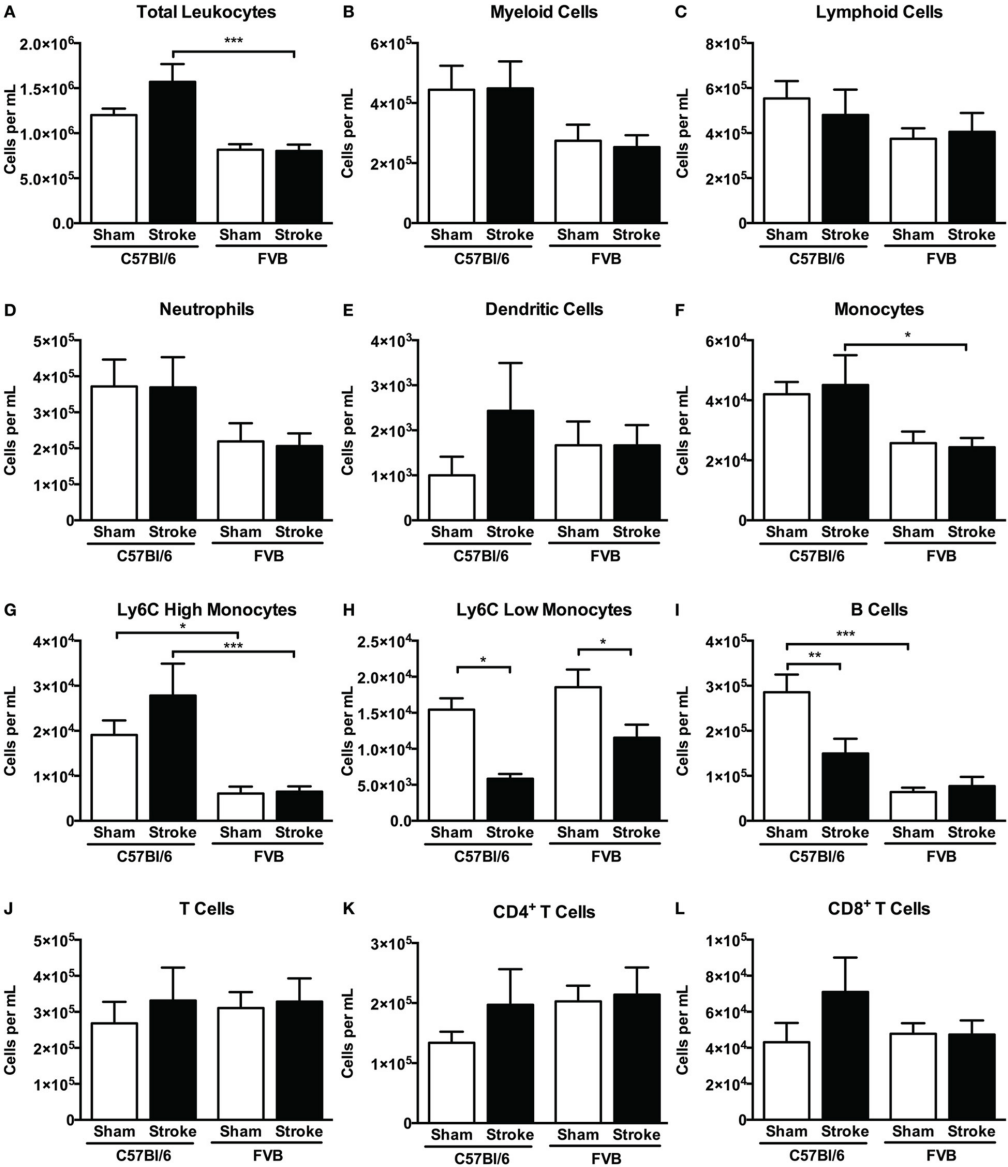
Quantification of (A) total leukocytes and (B–L) leukocyte subsets in blood 24 h after 1 h middle cerebral artery occlusion, compared with sham control animals of the same strain (total leukocytes *n* = 16–28, myeloid cells *n* = 9–17, lymphocytes *n* = 7–12; ^*^P < 0.05, ^**^P < 0.01, ^***^P < 0.001; One-Way ANOVA with Bonferroni post-tests). Data are mean ± s.e.m.

Overall, despite similar compositions of immune cells in the brains of C57Bl/6 and FVB mice following sham surgery, there were marked differences between strains after stroke with lymphoid:myeloid cells representing ~80:20 in total (Figure 6). While a similar ratio persisted in C57Bl/6 mice after ischemia, there was a markedly different leukocyte composition in FVB mice after stroke, with a reversal of the lymphoid:myeloid ratio to ~20:80 (Figure 6). There were few differences in blood composition of leukocytes between strains or after stroke (Figure 7).

### Splenic leukocyte numbers

**Figure 8:**
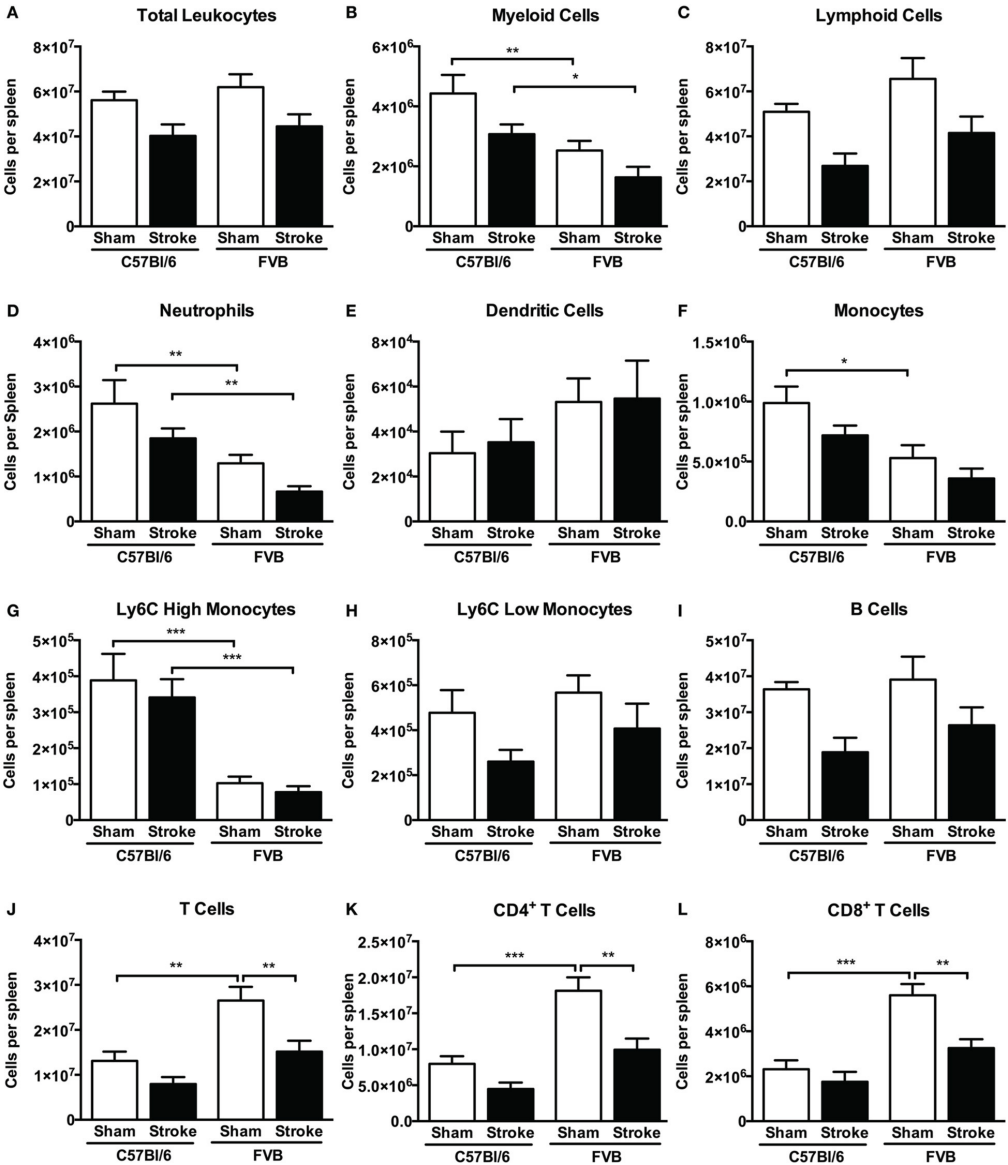
Quantification of (A) total leukocytes and (B–L) leukocyte subsets in spleen 24 h after 1 h middle cerebral artery occlusion, compared with sham control animals of the same strain (total leukocytes *n* = 17–29, myeloid cells *n* = 9–17, lymphocytes *n* = 6–12; ^*^P < 0.05, ^**^P < 0.01, ^***^P < 0.001; One-Way ANOVA with Bonferroni post-tests). Data are mean ± s.e.m.

At 24 h after stroke there was a tendency for a reduction in the total number of splenic leukocytes in both mouse strains (Figure 8A). There were fewer splenic myeloid cells in FVB vs. C57Bl/6 mice, due to lower numbers of neutrophils and Ly6C^high^ monocytes (Figures 8B,D,G), whereas there were higher numbers of CD4^+^, CD8^+^, and total T cells in that strain (Figures 8J–L). There was no significant reduction in any cell population (Figures 8C,E,F,H,I) except for 40–50% fewer T cells in FVB mice (Figures 8J–L). Consistent with these data, spleen weight was slightly reduced at 24 h after stroke in both strains, with the difference reaching statistical significance in FVB mice only (Supplementary Figure 2).

### Cytokine levels

Brain cytokine analysis at 24 h indicated that stroke resulted in substantially higher mean levels of IL-4, IL-6, TNF-α, IFN-γ, IL-10, and IL-17A in both C57Bl/6 and FVB mice (Supplementary Figure 3). There was a tendency for higher levels of IL-4, IL-6, TNF-α, and IFN-γ in the ischemic hemisphere of FVB than of C57Bl/6 (Supplementary Figure 3). There were no clear trends in cytokine levels in blood and spleen were generally similar in sham-operated mice of each strain, and there were no marked effects of stroke after 24 h in either strain (Supplementary Figures 4, 5).

## Discussion

There is growing evidence that T lymphocytes may influence the development of ischemic injury and functional deficit following experimental stroke (Iadecola and Anrather, 2011; Brait et al., 2012). For example, mice lacking T cells are reported to have smaller infarcts and improved functional outcome after focal ischemia compared to wild-type mice (Yilmaz et al., 2006; Hurn et al., 2007; Urra et al., 2009a; Kleinschnitz et al., 2013). Furthermore, there may be differential effects of CD4^+^ T cell subsets on stroke outcome, such as exacerbation by Th1 cells and amelioration by Th2 cells of brain infarct development and functional deficit (Xiong et al., 2011; Gu et al., 2012). There is also clinical evidence that single nucleotide polymorphisms in the genes of Th1 and Th2 cytokines, and molecules that regulate their transcription rate or their functionality, may predispose to immune responses of differing strength and thus contribute to the risk of stroke (Marousi et al., 2008).

Our study has examined representative mouse strains commonly accepted as Th1-dominant (C57Bl/6) and Th2-dominant (FVB) (Whitehead et al., 2003) to investigate stroke outcome at 24 h in prototypical Th1- vs. Th2-prone immune environments, respectively. Our data generally support the concept that Th1-prone immunity in C57Bl/6 results in a more severe functional outcome after stroke compared to Th2-prone FVB mice. For example, spleen levels of IL-4 and IL-10 were 3–4-fold higher in control FVB vs. C57Bl/6 mice. Yet, with no significant differences in cerebrovascular anatomy, degree of ischemic insult caused by MCA occlusion, and ultimately in the developed infarct size between the two mouse strains, there was a markedly different profile of immune cell infiltration in the ischemic hemispheres of C57Bl/6 and FVB mice.

Despite a similar immune cell composition in the brains of sham-operated C57Bl/6 and FVB mice, which comprised myeloid and lymphoid cells in a ~20:80 ratio, after ischemia there was an overall increase in infiltrating cell numbers in both strains whereby this ratio was preserved in C57Bl/6 but was converted to ~80:20 in FVB mice. The magnitude of total leukocyte infiltration into the brain of C57Bl/6 was approximately twice that observed in FVB, and these strain differences occurred in the absence of any notable stroke-related systemic differences such as cell numbers, cell composition or cytokine profile in either blood or spleen. Striking increases were noted to occur particularly in the number of innate immune cells such as neutrophils, macrophages and LyC6^+low^ monocytes infiltrating the ischemic FVB brain, whereas the most prominent increases in C57Bl/6 mice occurred in the numbers of infiltrating T and B lymphocytes and dendritic cells—key cells for adaptive immunity. These responses were associated with some strain differences in the mean levels of certain cytokines in the ischemic hemisphere, such as a tendency for larger amounts of both Th1 (IFN-γ, TNF-α) and Th2 (IL-4, IL-6) cytokines to be present in FVB than C57Bl/6 mice. However, it is not possible from the present data to discern whether these differences in brain cytokine levels may have been a cause or effect of the different post-ischemic immune cell profiles.

We found marked differences in neutrophil content of ischemic brains between FVB and C57Bl/6 mice. Clinical and experimental data suggest that neutrophils are the most abundant cell type in the brain after ischemia (Akopov et al., 1996; Gelderblom et al., 2009; Chu et al., 2014) and their accumulation is correlated with the severity of brain infarct and neurological deficit (Akopov et al., 1996). Activated neutrophils have been shown to promote the release of free radicals and cytokines, which further recruit leukocytes to the damaged area (Harris et al., 2005). However, it is controversial whether neutrophils contribute directly to secondary brain damage or have a mild neuroprotective role in cerebral ischemia. Animal studies have shown that increased infarct size is associated with neutrophil elimination (Takizawa et al., 2002) whilst others have shown that neutrophils may not contribute directly to infarct size (Beray-Berthat et al., 2003; Harris et al., 2005; Brait et al., 2011). Our data in FVB mice suggest that neutrophil number is not directly associated with infarct size in that a ~100-fold increase in brain infiltration after ischemia did not result in a bigger infarct size than in C57Bl/6 mice where the increase was markedly less. In previous study of parasite infection, neutrophils were found to play an early role in the induction of the Th2 response that develops in Th2-prone Balb/C mice but not in C57Bl/6 mice (Tacchini-Cottier et al., 2000). Thus, it is possible that the infiltration of neutrophils into the ischemic brains of FVB mice was associated with the induction of a less severe Th2-type immune response.

There were also markedly greater numbers of macrophages and Ly6C^low^ monocytes (the latter are considered to be anti-inflammatory) present in the FVB brains after cerebral ischemia. Macrophages and monocytes produce inflammatory cytokines and upregulate adhesion molecules in endothelial cells, thereby promoting neutrophil accumulation and migration (Chiba and Umegaki, 2013). Analogous to Th cells, macrophages are highly plastic cells and can polarize into two distinct activated macrophage subsets depending on the microenvironment (Kigerl et al., 2009). The classic or M1 activated cells are characterized by their capacity to present antigen, high production of nitric oxide and reactive oxygen species and of pro-inflammatory cytokines. In contrast, alternative or M2 activated cells are involved in scavenging of debris, angiogenesis, tissue remodeling and repair (Kigerl et al., 2009). Macrophages from Th1 strains (e.g., C57Bl/6, B10D2) are known to be more readily activated (e.g., to produce nitric oxide) than macrophages from Th2 strains (e.g., Balb/C, DBA/2) (Mills et al., 2000). Ly6C^+low^ monocytes are known to exhibit M2 characteristics (Geissmann et al., 2010). At the early stages following ischemic stroke, resident microglia and newly recruited macrophages appear to have a M2 phenotype that gradually transforms into an M1 phenotype in peri-infarct regions (Hu et al., 2012). It is possible that the greater number of macrophages and Ly6C^+low^ monocytes in FVB mice at 24 h after stroke represents more numerous M2-like cells contributing to a milder inflammatory environment in that strain. Further insight into the polarity of macrophages in FVB mice is needed.

We observed an increase in dendritic cells in the ischemic brain of C57Bl/6 mice at 24 h. Dendritic cells are involved in antigen presentation during immune cell activation and in the maintenance of peripheral tolerance through modulation of the immune response (Thompson and Thomas, 2002), but their role in outcome after cerebral ischemia is currently unclear.

There was a marked infiltration of lymphoid cells, particularly B and CD4^+^ T cells (i.e., Th cells), into the ischemic hemisphere of C57Bl/6 mice. T lymphocytes enter the brain by 24 h after ischemic stroke (Gelderblom et al., 2009; Kleinschnitz et al., 2013; Chu et al., 2014), and both CD4^+^ and CD8^+^ T cells contribute to post-ischemic injury in mice (Arumugam et al., 2005; Kleinschnitz et al., 2013). It is conceivable that the infiltration of CD4^+^ cells, which occurred selectively in C57Bl/6 mice following stroke, contributed to a more severe level of brain inflammation than in FVB mice despite a similar infarct volume. Tregs are a subset of CD4^+^ T cells that are reported to play a protective, immunomodulatory role in the brain over several days after stroke (Liesz et al., 2009), but a detrimental role during more acute conditions (Kleinschnitz et al., 2013). We found no significant changes after stroke in either CD8^+^ cells or CD4^+^CD25^+^ T cells, which will largely consist of Tregs.

Effects of B cells on stroke outcome are poorly understood. B cells can function as antigen-presenting cells to activate cytotoxic CD8^+^ T cells, but there is also data to suggest that an IL-10-producing subpopulation of regulatory B cells may limit injury in experimental stroke (Ren et al., 2011; Bodhankar et al., 2013). In addition, poor outcome in stroke patients is associated with reduced levels of circulating B cells (Urra et al., 2009b). Interestingly, we found that B cell infiltration into the brain following ischemia occurred selectively in C57Bl/6 mice (Figure 5A). Furthermore, whereas the number of circulating B cells was ~5-fold higher in control C57Bl/6 vs. FVB mice, stroke selectively reduced the number of B cells in the blood of the former strain (Figure 7I). Further study is necessary to clarify the importance of these strain differences in B cell number and distribution for stroke outcome.

In summary, we observed a profound difference in post-stroke functional outcome which was associated with a markedly contrasting number and composition of cells infiltrating the injured brain, despite similar systemic immune cell and cytokine profiles between the two mouse strains. The data therefore suggest that the nature of the inflammatory response to brain ischemia can vary considerably, and it may consequently impact the functional outcome independently of the volume of injured tissue. It would appear that the early infiltration into the ischemic brain tissue of certain innate/myeloid cell types, such as neutrophils, macrophages and Ly6C^+low^ monocytes, rather than cells of the adaptive immune system, such as B and T lymphocytes, may assist in achieving a milder level of functional deficit.

We acknowledge that while differences in biological responses between mouse strains that possess varying genetic and immunological profiles may provide a useful tool to gain some mechanistic insight into immune cell-mediated ischemic brain injury, studies of mice of the same genetic background are needed to provide definitive conclusions regarding these complex mechanisms. Moreover, if such findings are relevant for developing effective therapies for stroke patients, it will be interesting to determine whether individuals predisposed to Th2-prone immunity, including conditions such as asthma and allergy, might experience milder brain inflammation and functional deficit after stroke.

### Conflict of interest statement

The authors declare that the research was conducted in the absence of any commercial or financial relationships that could be construed as a potential conflict of interest.
