# Supplementary material for: Brain immune cell composition and functional outcome after cerebral ischemia: comparison of two mouse strains
Source: Front Cell Neurosci. 2014 Nov 19;8:365. doi: 10.3389/fncel.2014.00365 (PMC4237143; doi:10.3389/fncel.2014.00365)
Supplement: Supplementary file 1 [file Presentation1.PPTX]

## Slide 1
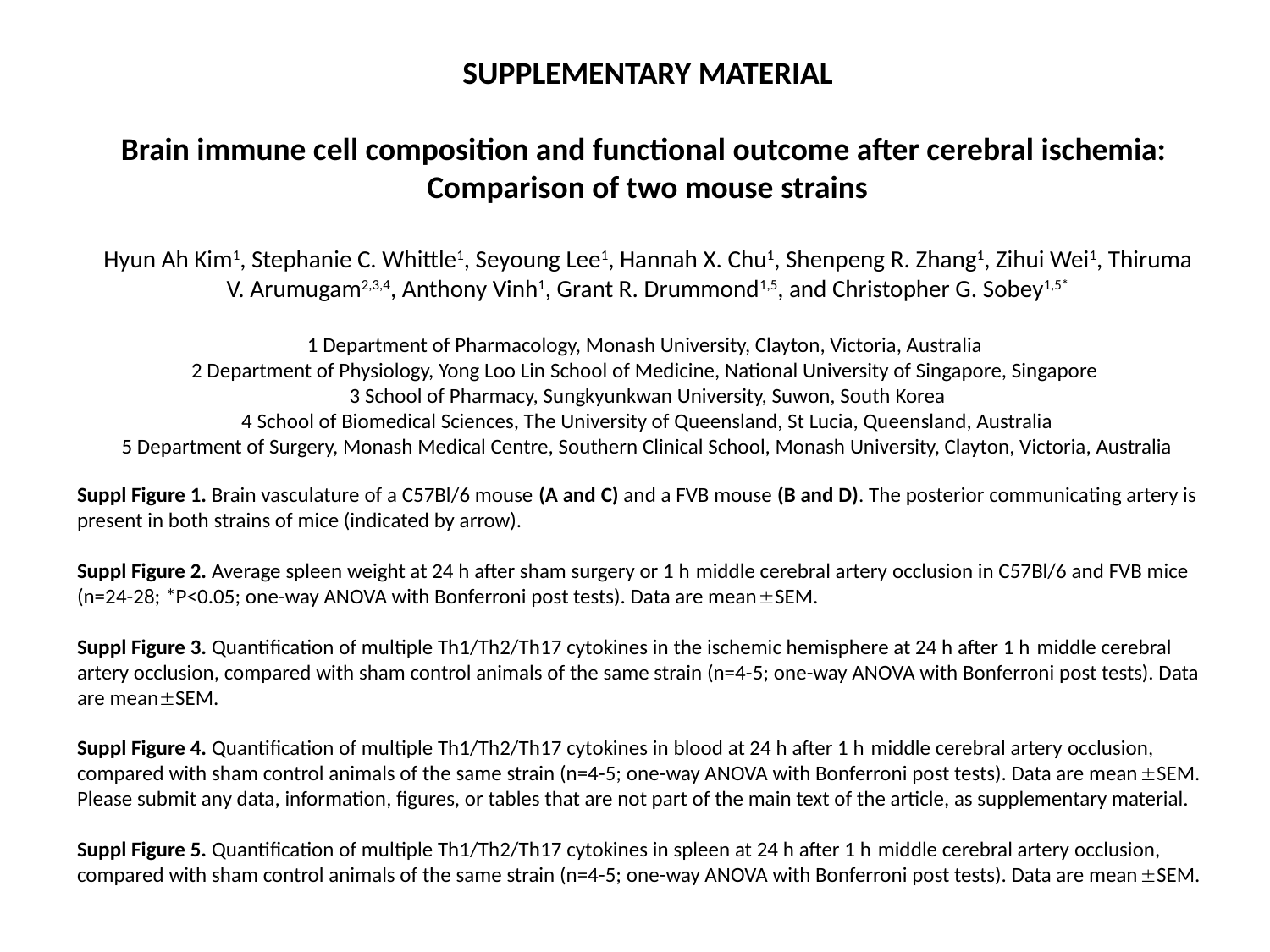

SUPPLEMENTARY MATERIAL
Brain immune cell composition and functional outcome after cerebral ischemia:
Comparison of two mouse strains
Hyun Ah Kim1, Stephanie C. Whittle1, Seyoung Lee1, Hannah X. Chu1, Shenpeng R. Zhang1, Zihui Wei1, Thiruma V. Arumugam2,3,4, Anthony Vinh1, Grant R. Drummond1,5, and Christopher G. Sobey1,5*
1 Department of Pharmacology, Monash University, Clayton, Victoria, Australia
2 Department of Physiology, Yong Loo Lin School of Medicine, National University of Singapore, Singapore
3 School of Pharmacy, Sungkyunkwan University, Suwon, South Korea
4 School of Biomedical Sciences, The University of Queensland, St Lucia, Queensland, Australia
5 Department of Surgery, Monash Medical Centre, Southern Clinical School, Monash University, Clayton, Victoria, Australia
Suppl Figure 1. Brain vasculature of a C57Bl/6 mouse (A and C) and a FVB mouse (B and D). The posterior communicating artery is present in both strains of mice (indicated by arrow).
Suppl Figure 2. Average spleen weight at 24 h after sham surgery or 1 h middle cerebral artery occlusion in C57Bl/6 and FVB mice (n=24-28; *P<0.05; one-way ANOVA with Bonferroni post tests). Data are mean±SEM.
Suppl Figure 3. Quantification of multiple Th1/Th2/Th17 cytokines in the ischemic hemisphere at 24 h after 1 h middle cerebral artery occlusion, compared with sham control animals of the same strain (n=4-5; one-way ANOVA with Bonferroni post tests). Data are mean±SEM.
Suppl Figure 4. Quantification of multiple Th1/Th2/Th17 cytokines in blood at 24 h after 1 h middle cerebral artery occlusion, compared with sham control animals of the same strain (n=4-5; one-way ANOVA with Bonferroni post tests). Data are mean±SEM. Please submit any data, information, figures, or tables that are not part of the main text of the article, as supplementary material.
Suppl Figure 5. Quantification of multiple Th1/Th2/Th17 cytokines in spleen at 24 h after 1 h middle cerebral artery occlusion, compared with sham control animals of the same strain (n=4-5; one-way ANOVA with Bonferroni post tests). Data are mean±SEM.

## Slide 2
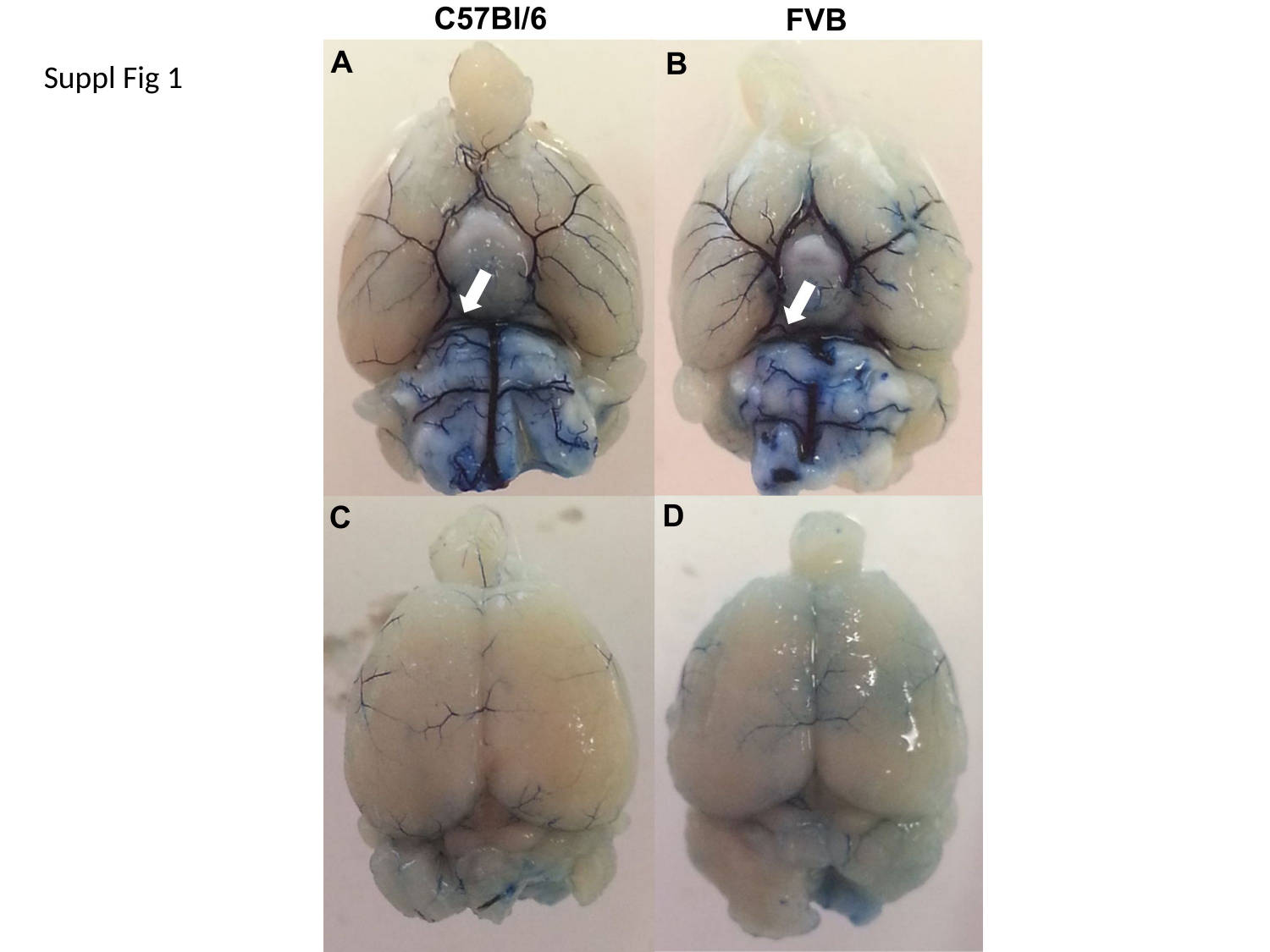

Suppl Fig 1

## Slide 3
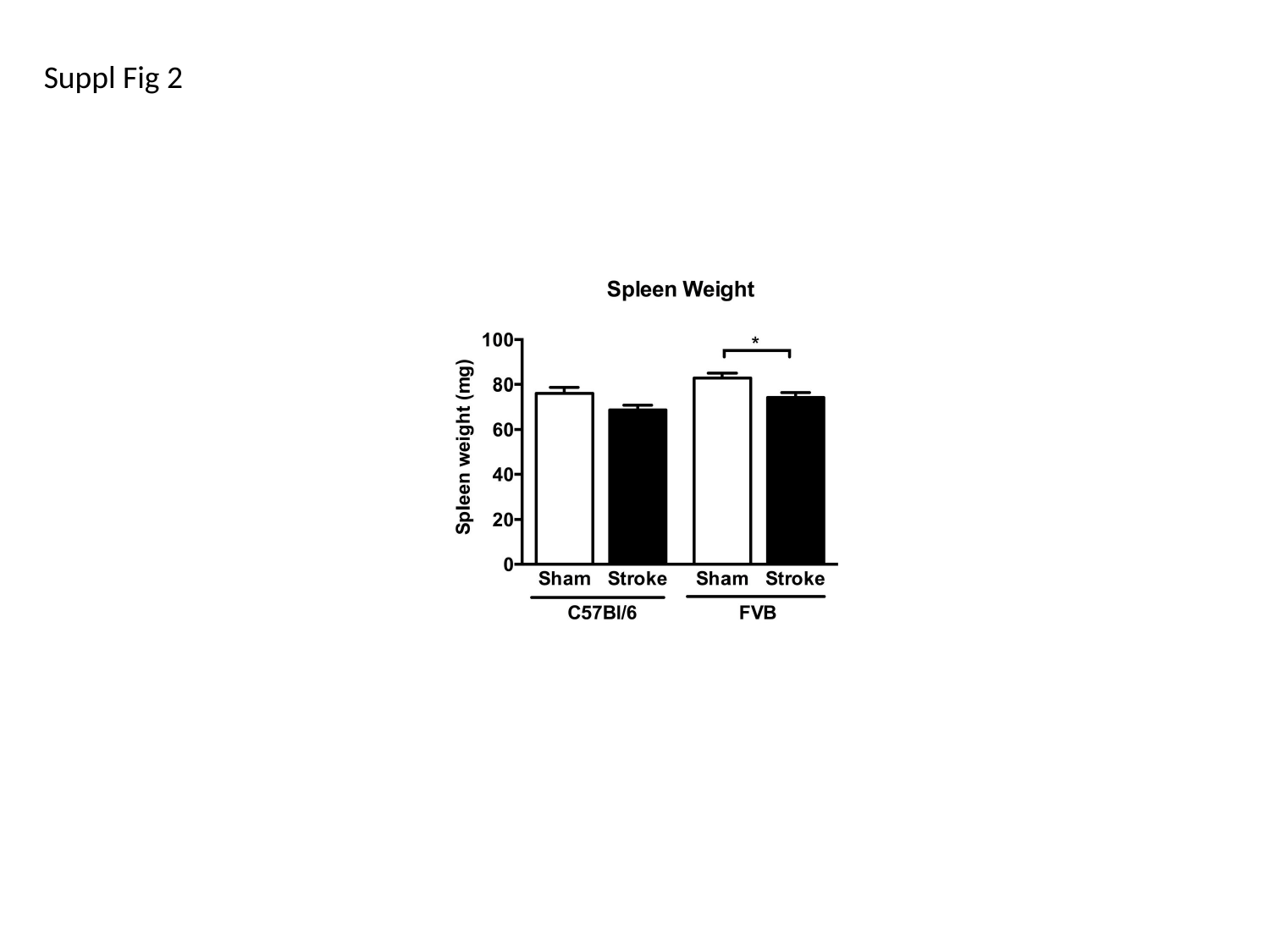

Suppl Fig 2

## Slide 4
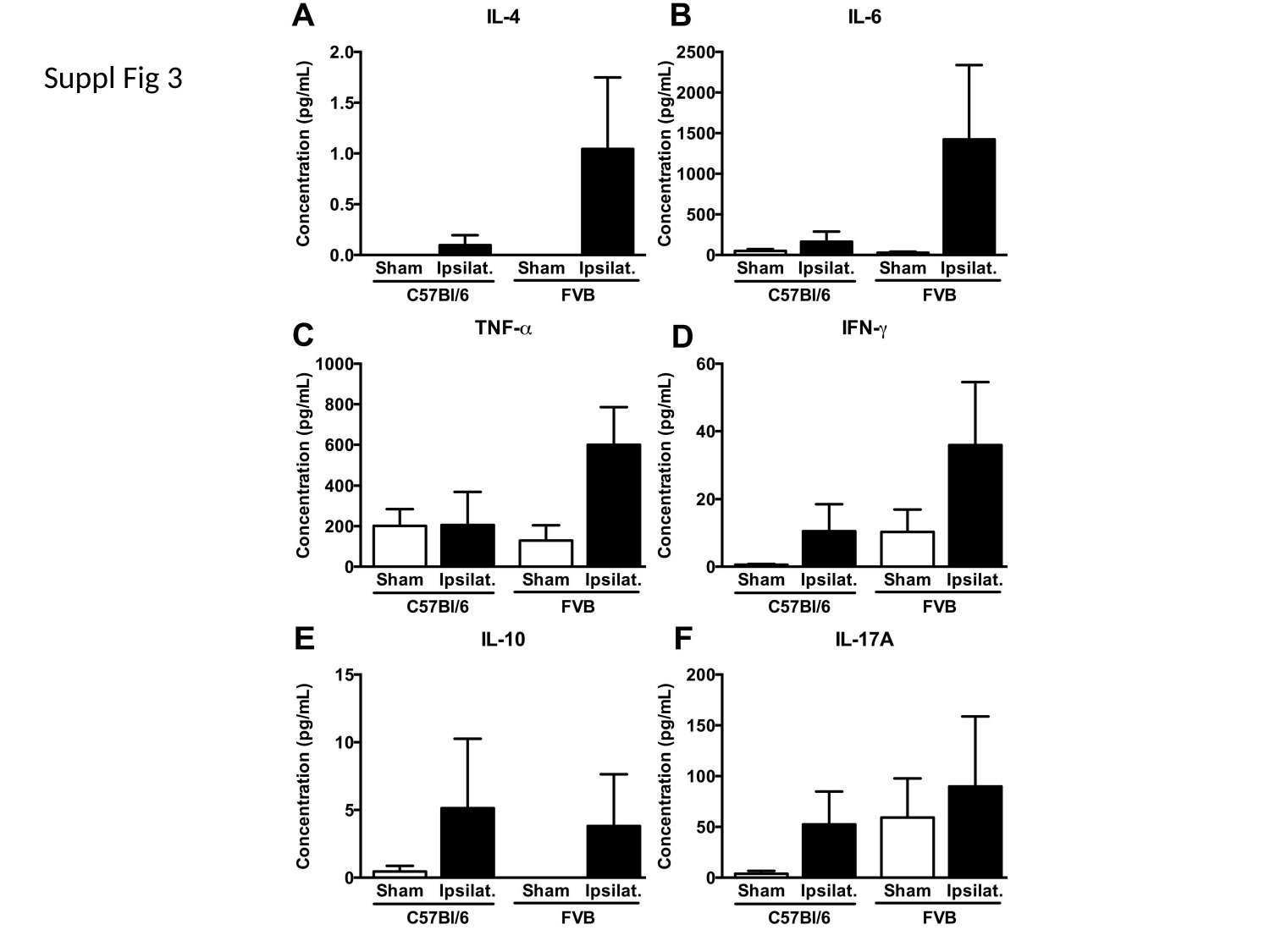

Suppl Fig 3

## Slide 5
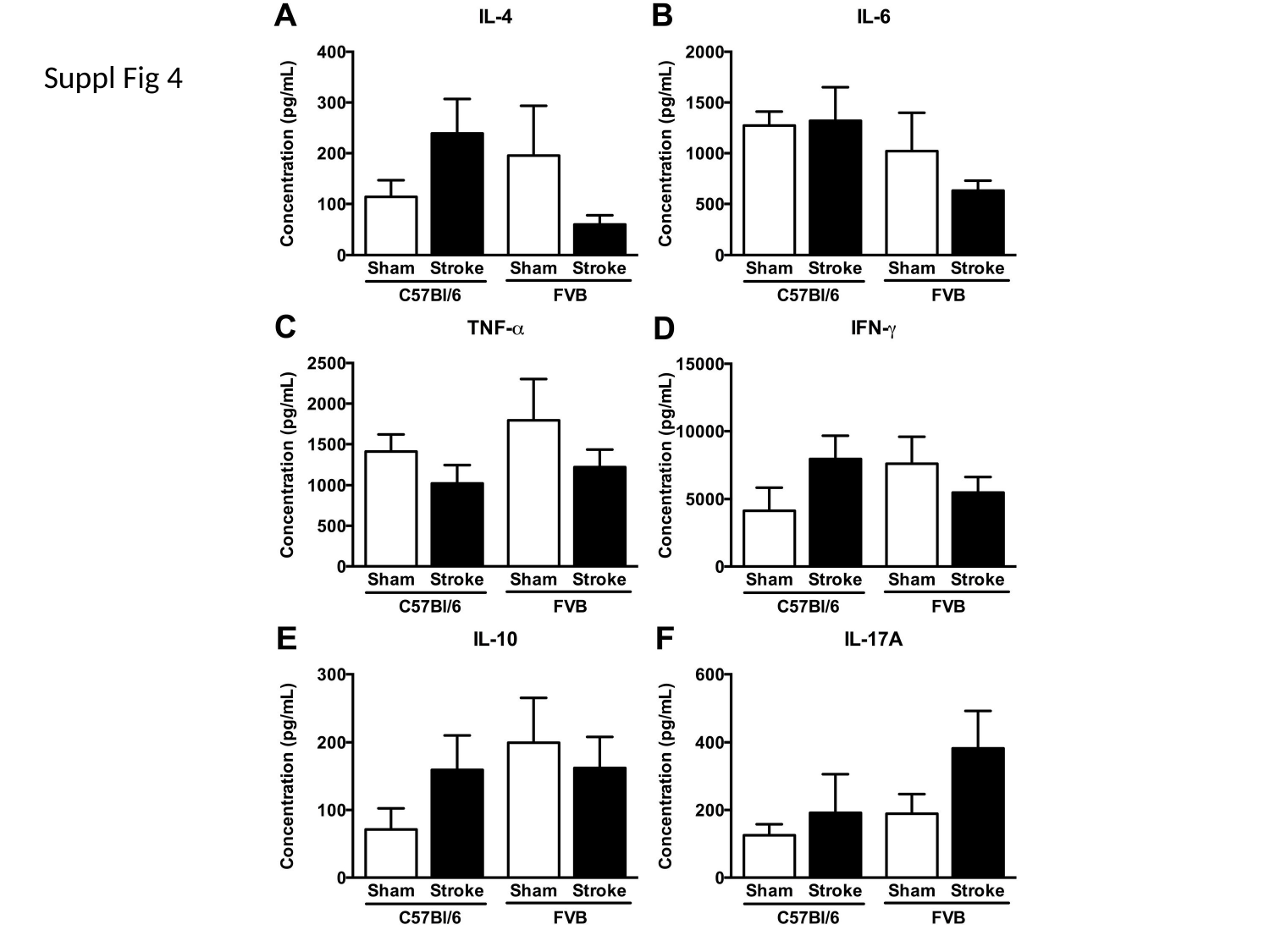

Suppl Fig 4

## Slide 6
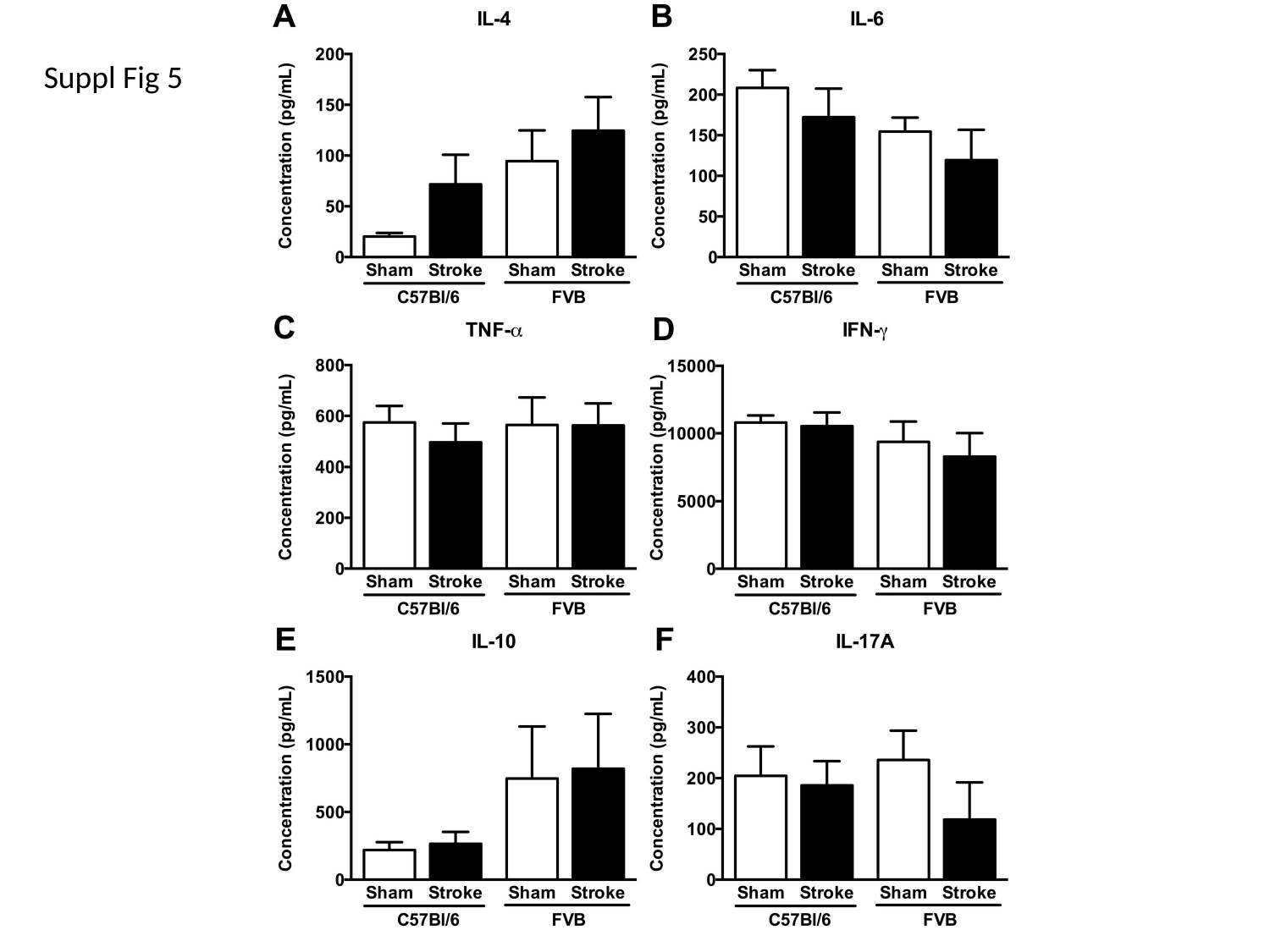

Suppl Fig 5
